# Comparative analysis of long-term oncologic outcomes for minimally invasive and open Ivor Lewis esophagectomy after neoadjuvant chemoradiation: a propensity score matched observational study

**DOI:** 10.1186/s13019-021-01728-z

**Published:** 2021-12-06

**Authors:** Robert E. Merritt, Peter J. Kneuertz, Mahmoud Abdel-Rasoul, Desmond M. D’Souza, Kyle A. Perry

**Affiliations:** 1 Division of Thoracic Surgery, The Ohio State University College of Medicine, The Ohio State University Wexner Medical Center, N847 Doan Hall, 410 West 10th Avenue, Columbus, OH 43210 USA; 2 Division of General and Gastrointestinal Surgery, The Ohio State University College of Medicine, The Ohio State University Wexner Medical Center, N847 Doan Hall, 410 West 10th Avenue, Columbus, OH 43210 USA; 3 Department of Biomedical Informatics, The Ohio State University College of Medicine, The Ohio State University Wexner Medical Center, N847 Doan Hall, 410 West 10th Avenue, Columbus, OH 43210 USA

**Keywords:** Esophageal carcinoma, Ivor Lewis esophagectomy, Neoadjuvant therapy, Minimally invasive esophagectomy

## Abstract

**Background:**

Locally advanced esophageal carcinoma is typically treated with neoadjuvant chemoradiation and esophagectomy (trimodality therapy). We compared the long-term oncologic outcomes of minimally invasive Ivor Lewis esophagectomy (M-ILE) cohort with a propensity score weighted cohort of open Ivor Lewis esophagectomy (O-ILE) cases after trimodality therapy.

**Methods:**

This is a retrospective review of 223 patients diagnosed with esophageal carcinoma who underwent neoadjuvant chemoradiation followed by M-ILE or O-ILE from April 2009 to February 2019. Inverse probability of treatment weighting (IPTW) adjustment was used to balance the baseline characteristics between study groups. Kaplan–Meier survival curves were calculated for overall survival and recurrence-free survival comparing the two groups. Multivariate Cox proportional hazards regression models were used to determine predictive variables for overall and recurrence-free survival.

**Results:**

The IPTW cohort included patients with esophageal carcinoma who underwent M-ILE (n = 142) or O-ILE (n = 68). The overall rate of postoperative adverse events was not significantly different after IPTW adjustment between the O-ILE and M-ILE trimodality groups (53.4% vs. 39.2%, *p* = 0.089). The 3-year overall survival (OS) for the M-ILE group was 59.4% (95% CI: 49.8–67.8) compared to 55.7% (95% CI: 39.2–69.4) for the O-ILE group (*p* = 0.670). The 3-year recurrence-free survival for the M-ILE group was 59.9% (95% CI: 50.2–68.2) compared to 61.6% (95% CI: 41.9–76.3) for the O-ILE group (*p* = 0.357). A complete response to neoadjuvant chemoradiation was significantly predictive of improved OS and RFS.

**Conclusion:**

The overall and recurrence-free survival rates for M-ILE were not significantly different from O-ILE for esophageal carcinoma after trimodality therapy. Complete response to neoadjuvant chemoradiation was predictive of improved overall and recurrence- free survival.

## Introduction

In the United States, there were an estimated 17,650 newly diagnosed cases of esophageal carcinoma in 2019 [1]. Neoadjuvant chemoradiation (CRT) and esophagectomy combined with comprehensive lymphadenectomy (trimodality therapy) is the current standard treatment for locally advanced esophageal carcinoma [2, 3]. The randomized Chemoradiotherapy for Oesophageal Cancer followed by Surgery Study (CROSS) Trial demonstrated improved overall survival and disease-free survival for patients who underwent preoperative chemoradiation followed by esophagectomy for locally advanced esophageal carcinoma [2]. Esophagectomy is the mainstay surgical treatment for esophageal carcinoma; however, the complex surgical procedure has been associated with significant morbidity and mortality [4]. Some reports have demonstrated evidence that preoperative chemoradiation may increase the incidence of postoperative complications after open esophagectomy [5, 6].

In recent years, minimally invasive techniques for Ivor Lewis esophagectomy were developed with the hope of minimizing postoperative morbidity and mortality. Luketich and colleagues reported a large single cohort series of over 1000 minimally invasive esophagectomy procedures that demonstrated a low postoperative mortality rate and a relatively low rate of postoperative pulmonary complications [7]. A randomized clinical trial also demonstrated decreased rates of postoperative pulmonary complications for minimally invasive transthoracic esophagectomy compared to the open approach [8]. In addition, a single center cohort study demonstrated fewer postoperative complications and a shorter hospital length of stay for minimally invasive esophagectomy compared to the open approach [9]. The previous comparative studies between minimally invasive Ivor Lewis esophagectomy (M-ILE) and open Ivor Lewis esophagectomy (O-ILE) have mostly focused on short-term clinical outcomes [10, 11]. However, the current gold standard for oncologic outcomes remains overall survival and recurrence-free survival. Only a few studies have evaluated the long-term oncologic results of minimally invasive esophagectomy compared to open esophagectomy [12–14]. In this study, we reported a propensity score adjusted comparison of the long-term oncologic outcomes (overall survival and recurrence-free survival) between M-ILE and O-ILE performed for patients with locally advanced esophageal carcinoma who completed neoadjuvant chemoradiation. We hypothesized that patients undergoing M-ILE after neoadjuvant chemoradiation for esophageal carcinoma will have equivalent long-term oncologic outcomes compared to a similar cohort of patients who underwent O-ILE.

## Methods

### Patient population

**Fig. 1:**
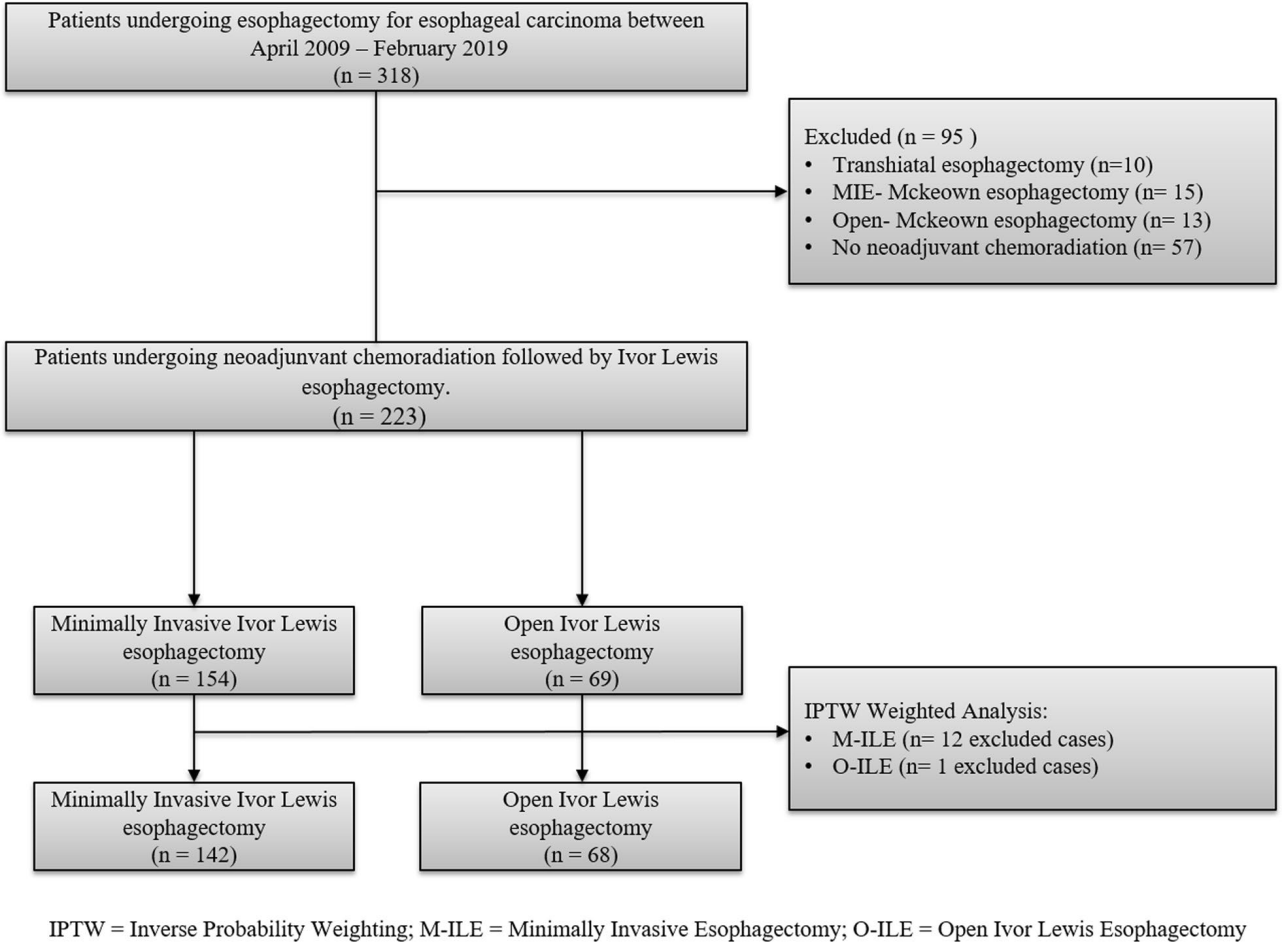
CONSORT diagram for patient selection and allocation

This study is a retrospective observational review of 223 patients who underwent neoadjuvant chemoradiation and Ivor Lewis esophagectomy for esophageal carcinoma on the General Thoracic Surgery Service at the Ohio State Wexner Medical Center between April 2009 and February 2019. The inverse probability of treatment weighting-adjusted (IPTW) cohort included patients with esophageal carcinoma who had undergone M-ILE (n = 142) or O-ILE (n = 68). The CONSORT diagram for the patient selection flowchart is shown in Fig. 1. Patients with adenocarcinoma or squamous cell carcinoma involving the mid-esophagus, distal third, or the gastroesophageal junction were included. The selection of the surgical approach was at the discretion of the treating surgeon. The study was approved by the Institutional Review Board and the requirement for informed consent was waived. The patients were clinically evaluated before the initiation of treatment with endoscopy, Computed Tomography (CT) scans, and clinical history and exam. Endoscopic Ultrasound and Positron Emission Tomography (PET) scans were performed for most patients prior to trimodality therapy (Table 1). The neoadjuvant treatment regimen consisted of weekly carboplatin with paclitaxel with concurrent radiotherapy (41.4, 45, or 50.4 Gray in fractions of 1.8 Gray). A PET CT scan was obtained 3–5 weeks after the completion of neoadjuvant chemoradiation to evaluate patients for clinical response and disease progression.

**Table 1:** Patient demographics

		Unadjusted			IPTW adjusted		
Variable	Levels	O-ILE N = 69	M-ILE N = 154	*p*-value	O-ILE N = 68	M-ILE N = 142	*p*-value
Age	Mean (Std Error)	62.36 (1.26)	62.31 (0.78)	0.968	61.31 (1.47)	62.51 (0.79)	0.813
Gender	Female	6 (8.7%)	24 (15.6%)	0.163	11.28%	10.40%	0.868
	Male	63 (91.3%)	130 (84.4%)		88.72%	89.60%	
Coronary artery disease	Yes	25 (36.2%)	44 (28.6%)	0.253	29.80%	30.81%	0.891
Diabetes	Yes	18 (26.1%)	42 (27.3%)	0.854	22.07%	25.44%	0.612
COPD	Yes	9 (13.0%)	14 (9.1%)	0.370	10.50%	9.90%	0.897
Cigarette smoking	Current smoker	14 (20.3%)	28 (18.2%)	0.825	14.65%	18.62%	0.729
	Never smoked	15 (21.7%)	39 (25.3%)		27.44%	23.13%	
	Past Smoker	40 (58.0%)	87 (56.5%)		57.91%	58.25%	
Body mass index	Mean (Std Error)	26.96 (0.72)	27.67 (0.45)	0.394	27.33 (0.66)	27.23 (0.44)	0.831
CT scan	Yes	58 (84.1%)	121 (78.6%)	0.341	84.45%	78.11%	0.350
Endoscopic ultrasound	Yes	58 (84.1%)	125 (81.2%)	0.603	72.74%	80.43%	0.321
PET scan	Yes	68 (98.6%)	154 (100.0%)	0.309	98.97%	100.00%	
ECOG status	0	40 (58.0%)	64 (41.6%)	0.023	45.00%	48.50%	0.669
	1/2	29 (42.0%)	90 (58.4%)		55.00%	51.50%	
Radiation dose	41 Gray	0 (0.0%)	1 (0.7%)	> 0.999	0.00%	0.70%	0.754
	45 Gray	13 (18.8%)	29 (18.8%)		17.70%	19.39%	
	50.4 Gray	56 (81.2%)	124 (80.5%)		82.30%	79.95%	
Time from CRT to surgery	Median Wks [IQR]	8.57 [6.86–10.29]	10.71 [9.43–13.43]	< 0.001	7.87 [5.83–9.81]	10.52 [9.13–13.12]	< 0.001

*IPTW* Inverse probability of treatment weighting, *O-ILE* Open Ivor Lewis esophagectomy, *M-ILE* Minimally invasive Ivor Lewis esophagectomy, *Std* Standard, *COPD* Chronic Obstructive Pulmonary Disease, *CT* Computed Tomography, *PET* Positron Emission Tomograpy, *CRT* Chemoradiation therapy, *IQR* Interquartile range

After a 6–12 week interval, patients underwent Ivor-Lewis esophagectomy with an open or minimally invasive approach. For the abdominal phase of the M-ILE, a complete laparoscopic or robotic gastric mobilization was performed with an en-bloc resection of the celiac and peri-gastric lymph nodes. For the thoracic phase of the M-ILE, a thoracoscopic or robotic mobilization of the esophagus was performed and an intrathoracic esophagogastric anastomosis, which was routinely covered with omentum or mediastinal pleura. The O-ILE was performed with a mid-line laparotomy incision and a posterior-lateral right thoracotomy incision. The general conduct of the operation is the same as described above for the M-ILE technique. The surgical specimens were reviewed and processed in a standardized manner. The pathologic stage after neoadjuvant therapy (ypTNM) was based on the 8^th^ edition of the AJCC/UICC staging manual [15]. The tumor regression grade is based on the Ryan scoring system [16]. Complete response (no viable cancer cells) is score 0; near complete response (single cancer cells or small clusters of cancer cells) is score 1; partial response (residual cancer with more than single cells) is score 2; poor or no response (extensive residual cancer with minimal evidence of tumor regression) is score 3. We also recorded perineural invasion, lympho-vascular invasion, tumor differentiation, signet ring cell features, and positive lymph nodes from the pathology reports.

### Outcome measures

The demographic data, clinical data, and perioperative outcomes were extracted from our institutional Society of Thoracic Surgery Database (Table 1). The clinical and pathologic stages were recorded using the American Joint Committee on Cancer, 8th edition, staging manual for esophageal carcinoma (Table 2). The primary outcomes were overall survival (OS) and recurrence-free survival (RFS). Overall survival was calculated as the date of surgery to the last follow up visit (censored) or death (event). A recurrence-free survival event was defined as the interval from surgery to either biopsy-proven or radiographic evidence of disease recurrence or death. The follow-up protocol included an initial postoperative visit within 2–3 weeks after esophagectomy and surveillance visits with a history and physical and contrast-enhanced computed tomography scans every 3–6 months for the first 3 years. Patients who did not have at least one follow-up at 3 months or greater were classified as lost to follow-up and were excluded from the study. The other oncologic endpoint included the total number of lymph nodes removed during esophagectomy. The secondary outcomes included the rate of postoperative adverse events, 30-day and 90-day mortality rate, length of stay, and 30-day readmission rates (Table 5). Multivariate Cox regression models were used to assess independent clinical and pathologic variables that predicted overall and recurrence-free survival after trimodality therapy.

**Table 2:** Staging and Pathologic Features

		Unadjusted			IPTW adjusted		
Variable	Levels	O-ILE N = 69	M-ILE N = 154	*p*-value	O-ILE N = 68	M-ILE N = 142	**p-value**
Clinical T-stage	1/2	22 (31.9%)	30 (19.5%)	0.043	21.00%	23.10%	0.731
	3	47 (68.1%)	124 (80.5%)		79.00%	76.90%	
Clinical N-stage	N0	16 (23.2%)	66 (42.9%)	0.001	41.46%	37.01%	0.868
	N1	50 (72.5%)	71 (46.1%)		51.38%	56.05%	
	N2	3 (4.3%)	17 (11.0%)		7.17%	6.95%	
Histology	Squamous Cell Carcinoma	3 (4.3%)	11 (7.1%)	0.426	3.07%	7.26%	0.166
	Adenocarcinoma	66 (95.7%)	143 (92.9%)		96.93%	92.74%	
Pathologic T-stage	T0	19 (27.5%)	41 (26.6%)	0.703	18.89%	27.22%	0.674
	T1a/T1b	12 (17.4%)	28 (18.2%)		17.78%	17.71%	
	T2	11 (15.9%)	34 (22.1%)		24.09%	22.50%	
	T3	27 (39.1%)	51 (33.1%)		39.24%	32.57%	
Pathologic N-Stage	N0/Nx	43 (62.3%)	99 (64.3%)	0.399	60.83%	66.18%	0.394
	N1	13 (18.8%)	37 (24.0%)		20.31%	22.92%	
	N2	7 (10.1%)	12 (7.8%)		10.22%	7.87%	
	N3	6 (8.7%)	6 (3.9%)		8.64%	3.03%	
Differentiation	Moderate	46 (66.7%)	74 (48.1%)	0.031	67.44%	50.42%	0.139
	Poor	21 (30.4%)	69 (44.8%)		28.77%	42.68%	
	Well	2 (2.9%)	11 (7.1%)		3.79%	6.90%	
Lymphovascular invasion	Yes	14 (20.3%)	42 (27.3%)	0.266	20.04%	27.24%	0.309
Perineural invasion	Yes	16 (23.2%)	30 (19.5%)	0.527	21.30%	19.64%	0.792
Signet ring cell features	Yes	4 (5.8%)	19 (12.3%)	0.138	9.93%	11.97%	0.741
Tumor regression grade	Complete response	18 (26.1%)	38 (24.7%)	0.043	18.60%	25.58%	0.274
	Near complete response	24 (34.8%)	58 (37.7%)		42.41%	36.52%	
	Partial response	11 (15.9%)	42 (27.3%)		20.61%	27.75%	
	No response	16 (23.2%)	16 (10.4%)		18.38%	10.14%	
Lymph nodes removed	Median [IQR]	18 [14–22]	17 [14–21]	0.956	16 [12–20]	17 [13–21]	0.822

*IPTW* Inverse probability of treatment weighting, *O-ILE* Open Ivor Lewis esophagectomy, *M-ILE* Minimally invasive Ivor Lewis esophagectomy, *IQR* Interquartile range

### Statistical analysis

The unadjusted baseline patient characteristics were compared between the M-ILE and O-ILE surgical approaches using chi-squared tests or Fisher exact tests where relevant for categorical variables and Student’s t-tests or Wilcoxon rank sum tests where relevant for continuous variables. The patient and disease characteristics were then balanced between the groups via propensity score methodology utilizing an inverse probability of treatment weighting (IPTW) technique to account for treatment selection bias [17]. The following differential variables were used to estimate the IPTW using binary logistic regression analysis: age, body mass index, gender, ECOG score, smoking status, coronary artery disease, COPD, and diabetes. IPTW-adjusted linear regression models were used to test for differences in the continuous variables. Overall survival and recurrence-free survival were estimated using IPTW-adjusted Kaplan–Meier analyses and multivariable Cox proportional hazards models including the same demographic characteristics used in the IPTW model listed above and compared using log-rank tests and Wald chi-square tests respectively.

IPTW-adjusted multivariable Cox proportional hazards models were fit for each of the overall and recurrence-free survival outcomes including patient baseline demographics and disease characteristic variables to assess potential confounding or effect modification of the relationship between surgery group and each of the OS and RFS outcomes. The proportional hazards assumption was assessed in each model by plotting the log of the negative log of the estimated survival density function vs log (time). Time dependent covariates were also tested and were not significant in the respective models. Hypothesis testing was conducted at an overall 5% type I error rate. The statistical analysis was designed and executed by an experienced biostatistician (MA) using the SAS, version 9.4 (SAS Institute, Cary NC).

## Results

A total of 318 patients who underwent esophagectomy for esophageal carcinoma were reviewed initially, but 38 patients were excluded from the study because they underwent different esophagectomy approaches other than the Ivor Lewis technique and 57 patients were excluded who did not receive neoadjuvant chemoradiation (Fig. 1). After IPTW adjustment, there were 142 patients in the M-ILE trimodality group and 68 patients in the O-ILE trimodality group who analyzed for the study. After the IPTW-adjusted analysis, the patient demographics (Table1) and the disease characteristics (Table 2) were better balanced between the M-ILE and O-ILE trimodality groups, with a similar distribution of age, gender, medical comorbidities, smoking status, BMI, radiation doses, histology, and clinical TNM stages. The R0 resection rates were 100% for both groups. The median number of lymph nodes removed during the M-ILE and O-ILE procedures after IPTW adjustment was 17 nodes [IQR: 13–21] and 16 nodes [IQR: 12–20], respectively (*p* = 0.822). The median follow-up period for the O-ILE trimodlaity group was 87.9 months ([IQR]: 63.3–107.5) and 46.6 months ([IQR]: 27.6–60.6) for the M-ILE trimodality group. The tumor differentiation, lymphovascular invasion, and signet ring cell features were present at similar rates in the pathologic resection specimen (Table 2). The tumor regression grades (complete, near complete, partial, or no response to neoadjuvant chemoradiation) were also similar between the study groups (Table 2).

**Fig. 2:**
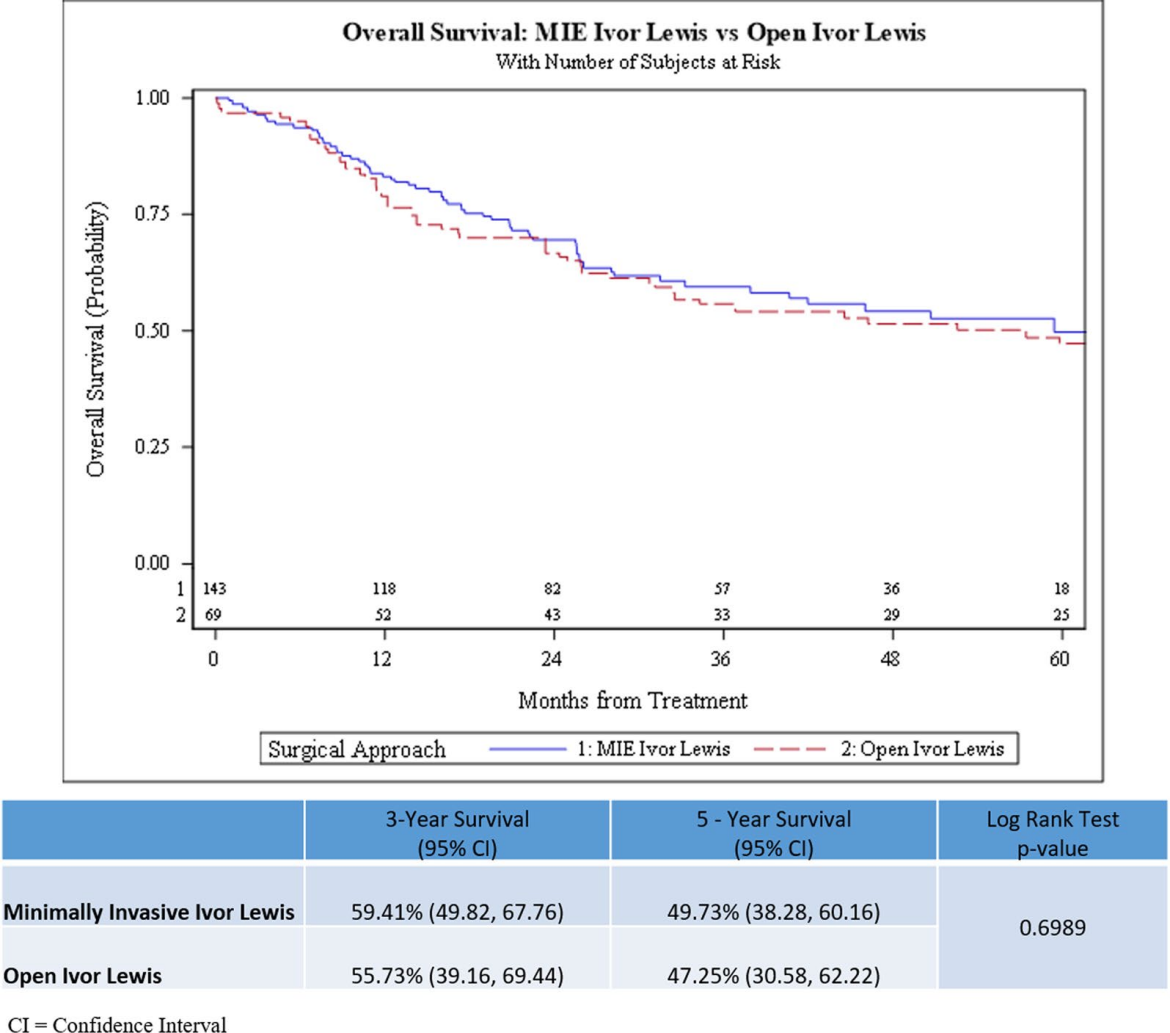
Kaplan Meier Curves for comparison of Overall Survival between minimally invasive (M-ILE) and open (O-ILE) Ivor Lewis esophagectomy after neoadjuvant chemoradiation

**Fig. 3:**
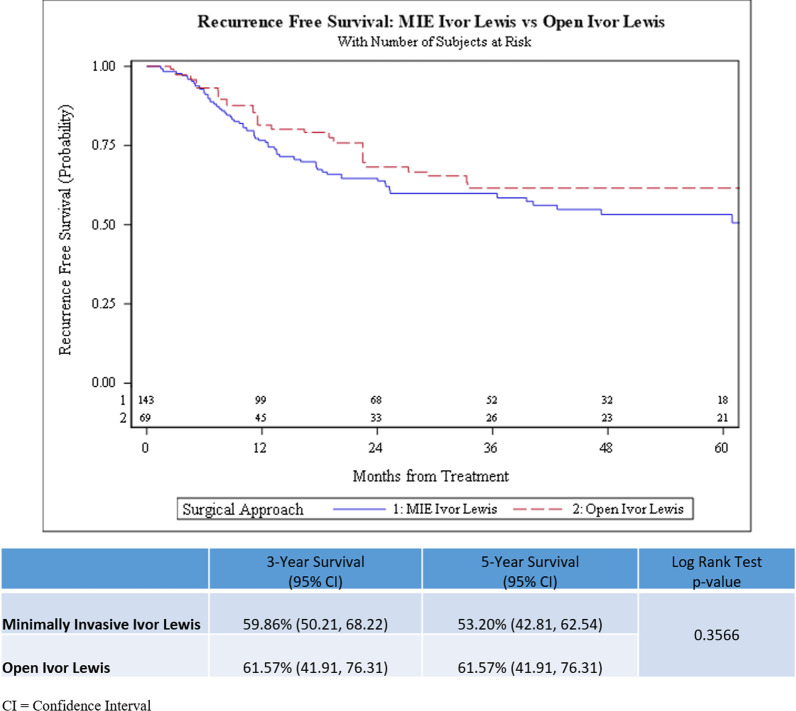
Kaplan Meier Curves for comparison of Recurrence-Free Survival between minimally invasive (M-ILE) and open (O-ILE) Ivor Lewis esophagectomy after neoadjuvant chemoradiation

There was no statistically significant difference observed in the overall survival (log-rank test *p* = 0.699) and recurrence-free survival (log-rank test *p* = 0.357) in patients who underwent M-ILE compared to O-ILE after neoadjuvant chemoradiation (Figs. 2 and 3). The 3-year OS rate was 59.41% (95% CI, 49.82–67.76%) in the M-ILE trimodality group and 55.73% (95% CI, 39.16–69.44%) in the O-ILE trimodality group. The 5-year OS rate was 49.73% (95% CI, 38.28–60.16%) in the M-ILE trimodality group and 47.25% (95% CI, 30.58–62.22%) in the O-ILE trimodality group. The 3-year RFS rate was 59.86% (95% CI, 50.21–68.22%) for the M-ILE trimodality group and 61.57% (95% CI, 41.91–76.31%) for the O-ILE trimodality group. The 5-year RFS rate was 53.20% (95% CI, 49.81–62.54%) in the M-ILE trimodality group and 61.57% (95% CI, 41.91–76.31%) in the O-ILE trimodality group. In an IPTW adjusted multivariable Cox regression model, the variables age, body mass index, clinical N-stage, and tumor regression grade were independent predictors of overall survival (Table 3). In the RFS multivariable model, clinical N-stage, signet ring cell features, and tumor regression grade were independent predictors of recurrence-free survival (Table 4).

**Table 3:** IPTW multivariable overall survival model

Parameter	HR (95% CI)	*p*-value
Study Group: MIE Ivor Lewis	0.95 (0.59, 1.53)	0.822
Study Group: Open Ivor Lewis	Reference	
Age (Years)	1.06 (1.03, 1.08)	< 0.0001
Body Mass Index (kg/m^2^)	0.95 (0.91, 0.98)	0.006
Clinical N stage: N0	0.41 (0.23, 0.73)	0.003
Clinical N stage: N1	0.83 (0.51, 1.37)	0.467
Clinical N stage: N2/N3	Reference	
Tumor regression grade: complete response	0.14 (0.07, 0.28)	< 0.0001
Tumor regression grade: near complete response	0.17 (0.09, 0.33)	< 0.0001
Tumor regression grade: partial response	0.39 (0.21, 0.74)	0.004
Tumor regression grade: no response	Reference	

*IPTW* Inverse probability of treatment weighting

**Table 4:** IPTW multivariable recurrence-free survival model

Parameter	HR (95% CI)	*p*-value
Study Group: MIE Ivor Lewis	1.64 (0.93, 2.87)	0.085
Study Group: Open Ivor Lewis	Reference	
Clinical N-Stage: N0	0.22 (0.11, 0.43)	< 0.0001
Clinical N-Stage: N1	0.53 (0.30, 0.92)	0.025
Clinical N-Stage: N2/N3	Reference	
Signet ring cell feature: no	0.50 (0.25, 0.99)	0.047
Signet ring cell feature: yes	Reference	
Anastomotic leak: no	0.54 (0.27, 1.10)	0.090
Anastomotic leak: yes	Reference	
Tumor regression grade: complete response	0.14 (0.06, 0.29)	< 0.0001
Tumor regression grade: near complete response	0.23 (0.12, 0.45)	< 0.0001
Tumor regression grade: partial response	0.30 (0.14, 0.62)	0.001
Tumor regression grade: no response	Reference	

*IPTW* Inverse probability of treatment weighting

The short-term postoperative surgical outcomes for esophagectomy by approach are listed in Table 5. After IPTW adjustment, the 30-day mortality rate was 0.55% for the M-ILE trimodality group and 3.24% for the O-ILE trimodality group (*p* = 0.084), which was not statistically significant. The 90-day mortality rates were similar between the two groups. The IPTW-adjusted median hospital length of stay was significantly shorter in the M-ILE trimodality group (7.5 days [IQR: 7–8]) versus (9 days [IQR: 7–13] in the O-ILE trimodality group (*p* = 0.01). The overall rate of postoperative adverse events was higher after IPTW adjustment in the O-ILE trimodality group, but the difference was not statistically significant (53.39% vs. 39.19%, *p* = 0.089). The anastomotic leak rate was lower in the M-ILE trimodality group compared to the O-ILE trimodality group [6.41% vs 12.63% (*p* = 0.228)], but the difference was not statistically significant. Respiratory failure and atelectasis requiring bronchoscopy occurred at significantly higher rates in the O-ILE trimodality group in the IPTW-adjusted analyses (Table 5). The IPTW-adjusted rate of unexpected ICU admission was significantly higher in the O-ILE trimodality group (31.46%) versus (9.75%) for the M-ILE trimodality group (*p* < 0.001). The 30-day readmission rates for the M-ILE and O-ILE trimodality groups were not significantly different (*p* = 0.293).

**Table 5:** Postoperative complications

	Unadjusted			IPTW adjusted		
Variable	O-ILE N = 69	M-ILE N = 154	*p*-value	O-ILE N = 68	M-ILE N = 142	*p*-value
Postoperative event occurred	36 (52.2%)	62 (40.3%)	0.098	53.39%	39.19%	0.089
Acute respiratory distress syndrome	0 (0.0%)	1 (0.6%)		0.00%	0.74%	
Acute myocardial infarction	0 (0.0%)	0 (0.0%)		0.00%	0.00%	
Air leak ≥ 5 Days	0 (0.0%)	0 (0.0%)		0.00%	0.00%	
Airway fistula	0 (0.0%)	5 (3.2%)		0.00%	3.08%	
Anastomotic leak	6 (8.7%)	9 (5.8%)	0.432	12.63%	6.41%	0.228
Anastomotic leak requiring repair	4 (5.8%)	5 (3.2%)	0.371	3.89%	3.47%	0.866
Anastomotic leak requiring stent	1 (1.4%)	5 (3.2%)	0.443	4.47%	3.56%	0.830
Conduit necrosis requiring surgery	2 (2.9%)	2 (1.3%)	0.405	2.13%	1.22%	0.576
Atelectasis requiring bronchoscopy	3 (4.3%)	2 (1.3%)	0.155	7.71%	0.78%	0.015
Atrial arrhythmia requiring treatment	10 (14.5%)	25 (16.2%)	0.741	16.67%	15.34%	0.828
Anastomotic stricture	4 (5.8%)	7 (4.5%)	0.690	5.06%	5.27%	0.948
Respiratory failure	11 (15.9%)	10 (6.5%)	0.026	18.12%	5.29%	0.006
Pneumonia	7 (10.1%)	8 (5.2%)	0.173	8.29%	5.43%	0.423
Empyema requiring treatment	1 (1.4%)	0 (0.0%)		0.93%	0.00%	
Ileus	2 (2.9%)	1 (0.6%)	0.178	5.00%	0.89%	0.119
Initial vent support 48 hour	4 (5.8%)	0 (0.0%)		5.49%	0.00%	
Pleural effusion	6 (8.7%)	3 (1.9%)	0.018	7.84%	2.46%	0.087
Postoperative blood transfusion	16 (23.2%)	10 (6.5%)	< 0.001	24.17%	6.28%	0.001
Pulmonary embolus	1 (1.4%)	1 (0.6%)	0.558	1.26%	0.58%	0.570
Renal failure	1 (1.4%)	1 (0.6%)	0.558	1.10%	0.00%	
Chylothorax	1 (1.4%)	0 (0.0%)		1.26%	0.00%	
Deep venous thrombosis	2 (2.9%)	4 (2.6%)	0.898	3.64%	2.75%	0.749
Delirium	7 (10.1%)	14 (9.1%)	0.803	9.36%	8.61%	0.859
Sepsis	3 (4.3%)	3 (1.9%)	0.306	3.45%	2.13%	0.555
Tracheostomy	2 (2.9%)	1 (0.6%)	0.178	3.07%	0.74%	0.214
Urinary tract infection	0 (0.0%)	1 (0.6%)		0.00%	0.82%	
Ventricular arrhythmia	2 (2.9%)	7 (4.5%)	0.564	4.81%	4.39%	0.915
Unexpected ICU admission	20 (29.0%)	13 (8.4%)	< 0.001	31.46%	6.86%	< 0.001
Hospital length of stay (Days): Median [IQR]	9 [7–13]	8 [7–9]	0.003	9 [7–13]	7.5 [7, 8]	0.010
Readmission within 30 days	6 (8.7%)	16 (10.4%)	0.695	5.88%	9.75%	0.293
Death 30 days after surgery	3 (4.4%)	1 (0.7%)	0.089	3.24%	0.55%	0.084
Death 90 days after surgery	3 (4.4%)	5 (3.3%)	0.705	3.24%	3.61%	0.883

*IPTW* Inverse probability of treatment weighting, *O-ILE* Open Ivor Lewis esophagectomy, *M-ILE* Minimally invasive Ivor Lewis esophagectomy, *IQR* Interquartile range

## Discussion

Trimodality therapy (neoadjuvant chemoradiation followed by esophagectomy) is the current standard treatment for locally advanced esophageal carcinoma [2]. Ivor Lewis esophagectomy has become the procedure of choice for the resection of esophageal carcinoma involving the distal third of the esophagus and the gastroesophageal junction at many medical centers [18]. In an attempt to minimize the perioperative morbidity associated with O-ILE, many high volume centers have developed minimally invasive Ivor Lewis esophagectomy techniques. While the short-term postoperative outcomes for M-ILE have been extensively compared to O-ILE in multiple cohort studies and a small randomized clinical trial, the long-term oncologic outcomes for M-ILE after nedoadjuvant chemoradiation have been examined in only a single known study. Tapias and colleagues compared the long-term oncologic and short-term operative outcomes of M-ILE (N = 56) and O-ILE (N = 74) after neoadjuvant therapy in a single institution cohort study [19]. In the study, the overall survival rates at 5 years were similar between the two groups (open: 61% versus MIE: 50%, *p* = 0.933). The complete resection rates and the number of lymph nodes were similar for the M-ILE and O-ILE groups as well.

Our report describes a series of M-ILE cases compared to a cohort of O-ILE cases performed after neoadjuvant chemoradiation at a single institution. This is the largest propensity score adjusted series comparing the long-term oncologic outcomes of M-ILE and O-ILE performed after the completion of neoadjuvant chemoradiation. The oncologic results of the M-ILE approach were comparable to the O-ILE technique after neoadjuvant chemoradiation in this report. The median number of lymph nodes that were dissected in the M-ILE group (17 nodes) were similar to the O-ILE group (16 nodes). Both surgical approaches yielded a median number of lymph nodes that were comparable to other reports [20, 21]. All of the patients in the O-ILE and M-ILE group underwent a complete R0 resection with negative surgical margins. The 3-year and 5-year overall survival and recurrence-free survival rates were not significantly different for the M-ILE trimodality group compared to the O-ILE trimodality group. This finding was consistent with the results of a small randomized phase III clinical trial comparing open transthoracic esophagectomy (McKeown and Ivor Lewis) to minimally invasive transthoracic esophagectomy after neoadjuvant therapy. The long-term follow up of the TIME (Traditional Invasive versus Minimally Invasive Esophagectomy) trial demonstrated no observed differences for 3-year overall survival (MIE: 50.5% versus Open: 40.4%, *p* = 0.207) and disease-free survival (MIE: 40.2% versus Open: 35.9%, *p* = 0.602) in patients who underwent minimally invasive transthoracic esophagectomy compared to open transthoracic esophagectomy [22].

In an IPTW adjusted multivariable Cox regression model, the variables age, body mass index, clinical N-stage, and tumor regression grade were independent predictors of overall survival. In the recurrence-free survival multivariable model, clinical N-stage, signet ring cell features, and tumor regression grade were independent predictors of recurrence-free survival. Tumor regression grade or the degree of pathologic response to neoadjuvant chemoradiation appeared to be a strong independent predictor for both overall and recurrence-free survival; whereas surgical approach (open versus MIE) did not demonstrate any prognostic significance. Takeda and colleagues performed a multivariable analysis on the varying degrees of pathologic response to neoadjuvant chemoradiation and demonstrated that tumor regression grade can reliably predict overall survival and systemic recurrence in patients with locally advanced esophageal cancer who were treated with trimodality therapy [23]. Similarly, Stiles and colleagues reviewed 238 patients who received neoadjuvant therapy followed by esophagectomy for locally advanced esophageal cancer. In a multivariable model, a poor clinical response to neoadjuvant therapy (HR 2.77; 95% CI: 1.14–7.15) was highly predictive of early mortality within a year of trimodality therapy [24]. These findings indicate that the tumor regression grade or response to neoadjuvant therapy has more prognostic significance than the surgical approach for esophagectomy.

Propensity score weighting and multivariable regression analysis allowed us to statistically balance baseline differences and control for confounders in the short-term perioperative outcomes and the long-term oncologic outcomes. Nonetheless, the present study has several limitations which should be considered when interpreting the results of study. The selection process for the surgical approach was determined by the surgeon based on individual preference and could include factors that were not captured in the study. Given the retrospective observational study design, we could not completely eliminate unmeasured selection bias; therefore, the results of the study are not generalizable. The follow up time for the M-ILE group was shorter, which decreased the accuracy of the survival data after the 3-year time point. However, the surveillance data for the first three years was robust and likely captured most of the recurrence cases and deaths.

## Conclusions

In conclusion, OS and RFS rates were not significantly different between the M-ILE and O-ILE trimodality groups. Our analysis demonstrated that OS and RFS for locally advanced esophageal caracinoma are more likely determined by the degree of pathologic response to neoadjuvant chemoradiation as opposed to surgical approach for esophagectomy. The short-term surgical outcomes, such as respiratory failure and hospital length of stay, for patients undergoing M-ILE were significantly improved compared to patients undergoing O-ILE after IPTW adjustment for baseline demographic covariates. These results support the use of minimally invasive Ivor Lewis esophagectomy after neoadjuvant chemoradiation for surgical treatment of local advanced esophageal carcinoma at our institution.

## Data Availability

The dataset analyzed during the current study are not publicly available due to institutional restrictions on sharing patient data but can be made available from the corresponding author on reasonable request. Most of the data is included in the tables and figures.

## Abbreviations

AJCC: American Joint Committee on Cancer
BMI: Body Mass Index
CI: Confidence Interval
CRT: Chemoradiation
CROSS: Chemoradiotherapy for Oesophageal Cancer followed by Surgery Study
COPD: Chronic obstructive pulmonary disease
CT: Computed tomography
ECOG: Eastern Cooperative Oncology Group
IPTW: Inverse probability of treatment weighting
IQR: Interquartile range
M-ILE: Minimally invasive Ivor Lewis Esopahgectomy
O-ILE: Open Ivor Lewis Esophagectomy
OS: Overall survival
PET: Positron emission tomography
RFS: Recurrence-free survival
SAS: Software analytics and solutions
TNM: Tumor-node-metastasis
UICC: Union of International Cancer Control
